# Effects of Open Versus Closed Skill Exercise on Cognitive Function: A Systematic Review

**DOI:** 10.3389/fpsyg.2019.01707

**Published:** 2019-07-27

**Authors:** Qian Gu, Liye Zou, Paul D. Loprinzi, Minghui Quan, Tao Huang

**Affiliations:** ^1^Department of Physical Education, Shanghai Jiao Tong University, Shanghai, China; ^2^Lifestyle (Mind-Body Movement) Research Center, College of Psychology, Shenzhen University, Shenzhen, China; ^3^Exercise & Memory Laboratory, Department of Health, Exercise Science and Recreation Management, The University of Mississippi, University, MS, United States; ^4^School of Kinesiology, Shanghai University of Sport, Shanghai, China

**Keywords:** motor skill, open skill exercise, closed skill exercise, cognition, executive function

## Abstract

**Background:**

Exercise modes can be divided into open skill exercise (OSE) and closed skill exercise (CSE). While research has shown that these two exercise modes may have different effects on cognitive function, this possibility has not been systematically reviewed.

**Objective:**

The purpose of the present review was to objectively evaluate the research literature regarding the effects of OSE versus CSE on cognitive function.

**Methods:**

Six electronic databases (Web of Science, EMBASE, Google Scholar, PubMed, PsycINFO, and SPORTDiscus) were searched from inception dates to December 2018 for studies examining the associations of OSE and CSE with cognitive function. The literature searches were conducted using the combinations of two groups of relevant search items related to exercise modes (i.e., OSE and CSE) and cognitive function. Articles were limited to human studies in all age groups. Both intervention and observational studies with full text published in English-language peer-reviewed journals were considered eligible. The search process, study selection, data extraction, and study quality assessment were carried out independently by two researchers.

**Results:**

A total of 1,573 articles were identified. Fourteen observational and five intervention studies met the inclusion criteria. Twelve of the 14 observational studies found that OSE benefits cognitive function, and seven of these 14 observational studies supported superior effects of OSE compared with CSE for enhancing cognitive function. Three of the five intervention studies found that OSE (versus CSE) led to greater improvements in cognitive function in both children and older adults.

**Conclusion:**

Although the majority of studies in this review were observational cross-sectional designs, the review tends to support that OSE is more effective for improving some aspects of cognitive function compared with CSE. More rigorous randomized control trials with long-term follow-ups are needed in order to confirm these differential cognitive effects of the two exercise modes.

## Introduction

The beneficial effects of physical activity and exercise on physical health have been well-documented among all age groups (Booth et al., 2012; Hills et al., 2015), and an increasing number of researchers have recently paid great attention to investigating further associations between exercise and cognitive function (Lin et al., 2018; Pedersen, 2019; Stern et al., 2019). Cognitive functions refer to mental processes of obtaining knowledge and understanding through thought, experience, and the senses, including perception, attention, visual and spatial processing, language, memory, executive functions, etc. (Lezak et al., 2012). Executive function, also termed cognitive control, refers to higher-order, self-regulatory cognitive processes that aid in the monitoring and control of thought and action (Carlson, 2005). It encompasses working memory, inhibitory control, cognitive flexibility, reasoning, planning and problem solving, etc. (Diamond, 2013). Executive function plays a crucial role in daily life and it has attracted much attention in current research. Although existing evidence has shown that physical fitness and exercise have important relationships with various aspects of cognitive functions (Kramer and Erickson, 2007; Aberg et al., 2009; Chaddock et al., 2011), studies tend to suggest that the beneficial effects of exercise are larger and more evident for executive function (Kramer and Erickson, 2007; Chaddock et al., 2011). For example, a higher level of physical fitness has been associated with better executive function and academic performance in children and adolescents (Huang et al., 2015; Marques et al., 2018; Westfall et al., 2018). Physical exercise intervention programs can enhance children’s executive function performances as measured by inhibition and cognitive flexibility tasks (Hillman et al., 2014). Additionally, people who exercised regularly have demonstrated slower cognitive declines and a lower risk of developing dementia (Middleton et al., 2010; Zotcheva et al., 2018). Well-designed randomized controlled trials have also provided compelling evidence that physical exercise interventions can improve executive function and spatial memory in older adults (Kramer et al., 1999; Erickson et al., 2011). Furthermore, current evidence suggests that different types of physical exercise may exert differential influences on cognitive function and mental health (Tsai et al., 2012; Tsai and Wang, 2015; Chekroud et al., 2018). Yet, there remains some controversy regarding what types of physical exercises may be more effective for improving cognitive function.

Recently, studies have suggested that the extent of improvements in cognitive function through physical exercise may be related to the motor movement characteristics of the activities involved (Guo et al., 2016; Chang et al., 2017; Cho et al., 2017). According to the effects of environment on motor skills, motor skills can be divided into open and closed skills (Knapp, 1967). Open skills are performed in a dynamic and changing environment, while closed skills take place in a predictable and static environment (Galligan, 2000). Accordingly, exercise modes can be classified into open skill exercise (OSE) and closed skill exercise (CSE) (Di Russo et al., 2010; Dai et al., 2013; Tsai and Wang, 2015; Tsai et al., 2016, 2017). OSEs (e.g., table tennis, tennis, squash, basketball, or boxing) involve unpredictable environments, active decision making, and ongoing adaptability in which participants must alter responses to randomly occurring external stimuli (Brady, 1995; Di Russo et al., 2010; Wang et al., 2013a). OSEs are predominantly perceptual and externally paced. In contrast, CSEs (e.g., running, swimming, cycling, golf, or archery) are performed in a relatively stable and predictable environment in which motor movements follow set patterns. CSE skills tend to be self-paced, as there are fewer cognitive demands and decision-making requirements (Brady, 1995; Di Russo et al., 2010; Wang et al., 2013a). In the context of this conceptual framework, researchers have investigated the associations of OSE and CSE with cognitive function among participants in different age groups. Some studies have shown that OSE participants performed better in some aspects of executive function (e.g., inhibitory control and cognitive flexibility) than CSE participants (Giglia et al., 2011; Dai et al., 2013; Wang et al., 2013a). In contrast, some studies reported that the cognitive effects of OSE and CSE did not differ (Chang et al., 2017; Chueh et al., 2017; Becker et al., 2018).

Despite the rapid expansion of interest in this topic, there has been no systematic review of existing literature that has critically evaluated the differential effects of OSE versus CSE on cognitive function across the lifespan. Given a lack of clarity regarding suspected differences in the benefits of these exercise modes for benefiting cognitive function, we undertook the current systematic review of intervention (including acute exercise and chronic exercise) and observational research to date.

## Materials and Methods

This systematic review was conducted according to the Preferred Reporting Items for Systematic Reviews and Meta-Analyses (PRISMA) (Moher et al., 2009). Notably, as demonstrated hereafter, there was considerable heterogeneity across the studies, regarding study design and participant characteristics. As such, a meta-analysis was not conducted with this systematic review.

### Literature Searches

We began with a computerized search of six electronic databases (Web of Science, EMBASE, Google Scholar, PubMed, PsycINFO, and SPORTDiscus) for all research in these databases up to December 2018. Articles were limited to human studies in all age groups. There was no restriction on publication year. We used the combinations of the following two groups of retrieval terms: (a) OSE and CSE, feedback exercise and non-feedback exercise, open loop exercise and closed loop exercise, and planned exercise and incidental exercise, and (b) cognition, cognitive function, executive function, working memory, memory, inhibitory control, and cognitive flexibility. Each (a) item was combined with all (b) items during the search process. In order to exclude duplicate or apparently irrelevant studies, the authors next screened all retrieved titles. From this shorter list, two authors (QG and TH) independently reviewed the abstracts of each remaining study. Having further reduced number of the articles in this manner, the two authors (QG and TH) then independently screened the full text of the remaining studies, using predetermined inclusion and exclusion criteria (see below for details). Disagreements were discussed in all cases until a consensus was reached among the authors. The review authors then searched the bibliographies of all included articles in the same fashion as outlined above to further ensure that relevant articles had been captured.

### Inclusion/Exclusion Criteria for Study Selection

The identified studies were initially screened by two authors (QG and TH) to determine whether they met our inclusion criteria as follows: (a) intervention [assigned into either an experimental arm (OSE or CSE) or control arm] and observational studies with full text published in English-language peer-reviewed journals; (b) both OSE (a type of exercise is performed in an unpredictable environment, where the exerciser is not the one who decides when the skill and movement need to be executed such as some team-based sports and racket sports) and CSE (a type of exercise is performed in a relatively stable environment, where the exerciser is able to dictate when he or she starts to perform the motor skill) have to be clearly defined and simultaneously examined in the study; (c) study outcomes must include at least one measurement of any aspects of cognitive function (e.g., perception, attention, visual and spatial processing, language, memory, and executive functions). It is worth emphasizing that the participants’ ages, gender, race/ethnicity, etc., were not restricted in this systematic review in order to gain a comprehensive understanding of this new topic based on the inclusion of all relevant articles. This review excluded studies which applied other combined interventions such as OSE or CSE plus a nutrition program. The studies were finally included when a consensus was reached by two authors.

### Data Collection

Information regarding publication year, participant characteristics, location, intervention program, exercise experience, educational level, outcomes (assessment instruments) and study results were independently extracted by two authors (QG and TH) for later analysis and presentation.

### Methodological Quality

Methodological quality was assessed independently by two authors (QG and TH). Any discrepancies in the ratings of the methodological quality were settled by discussion, based on the scoring criteria of the two assessment instruments, among the authors until a consensus was reached. The two authors independently assessed the methodological quality of the intervention studies using the Physiotherapy Evidence Database (PEDro) scale (Elkins et al., 2013). The PEDro scale consists of 11 items, namely eligibility criteria, randomization, allocation concealment, baseline equivalence, blinding of the instructor, blinding of participants, blinding of outcome assessors, retention rate of ≥ 85%, intention-to-treat analysis, between-group comparison, and point measures and measures of variability. A maximum of 11 points can be obtained (clear description = 1 and unclear description = 0) (Elkins et al., 2013). The methodological quality of observational studies was assessed using the evaluation tool developed by Fuzeki et al. (2017) and Engeroff et al. (2018). It consists of five components (12 items in total), namely the assessment of study purpose, study design and methods, statistical methods, results, and discussion. The 12 items of the assessment tool are listed in Supplementary Table 1. A maximum of 12 points can be obtained. According to Fuzeki et al. (2017), the quality of studies can be divided into three categories (≥10 points as high quality; 6–9 points as moderate quality; and <6 points as low quality).

## Results

### Study Selection

**FIGURE 1:**
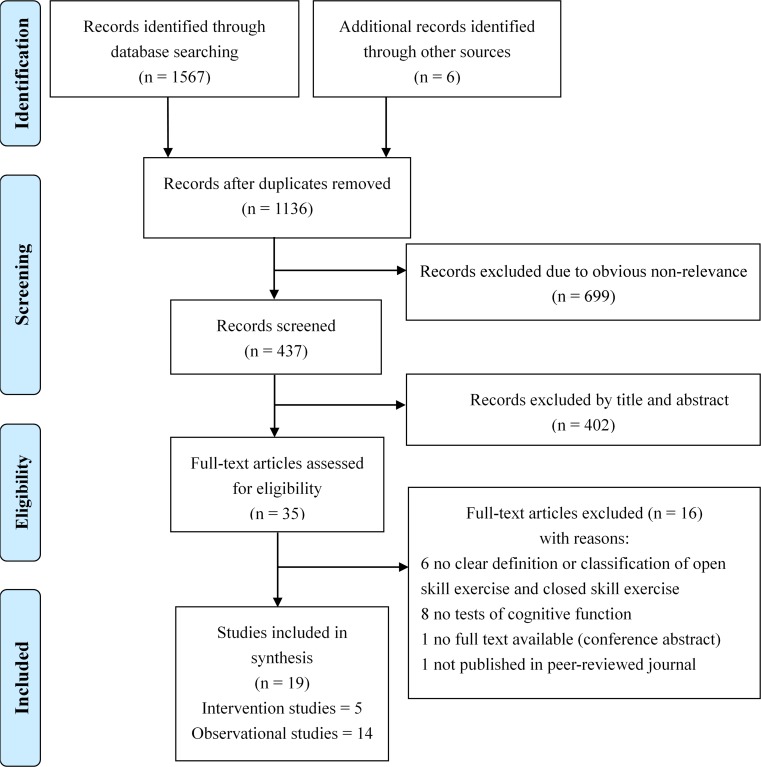
Flow diagram of each stage of the study selection.

A flowchart of our study selection process is shown in Figure 1. The search strategy first identified 1567 potential articles from the six electronic databases as well as six additional records that were identified through checking the references in the most relevant studies. After removing duplicates and irrelevant articles, 437 articles remained for screening via title and abstract. Of these, 35 were identified as potentially relevant. After independently evaluating the full text of these 35 articles using the predefined inclusion criteria by the two reviewer authors (QG and TH), we excluded 16, leaving 19 studies eligible for this systematic review. Fourteen studies were observational in design (Giglia et al., 2011; Dai et al., 2013; Wang et al., 2013a, b; Huang C.J. et al., 2014; Jacobson and Matthaeus, 2014; Tsai and Wang, 2015; Guo et al., 2016; Tsai et al., 2016; Chang et al., 2017; Chueh et al., 2017; Yu et al., 2017; Becker et al., 2018; Li et al., 2018) and five were intervention studies (Crova et al., 2014; Schmidt et al., 2015; O’Brien et al., 2017; Tsai et al., 2017; Hung et al., 2018). Based on the pretermined inclusion criteria, study selection was independently performed by two raters and the agreement score was 18 out of 19. To resolve this disagreement on this study, a third author was invited to discuss and finally reach a consensus.

### Characteristics of Included Studies

As noted above, five of the 19 included studies were intervention studies (including two acute intervention studies) (Crova et al., 2014; Schmidt et al., 2015; O’Brien et al., 2017; Tsai et al., 2017; Hung et al., 2018). These five intervention studies included two involving children (Crova et al., 2014; Schmidt et al., 2015), one involving young adults (aged 18–35 years) (Hung et al., 2018) and two involving older adults (aged older than 55 years) (O’Brien et al., 2017; Tsai et al., 2017). Within the two studies involving children (Crova et al., 2014; Schmidt et al., 2015), OSE intervention programs were administrated through physical education classes. The other three studies (O’Brien et al., 2017; Tsai et al., 2017; Hung et al., 2018) were conducted in laboratory settings and the exercise interventions were supervised.

Among the 14 observational studies (Giglia et al., 2011; Dai et al., 2013; Wang et al., 2013a, b; Huang C.J. et al., 2014; Jacobson and Matthaeus, 2014; Tsai and Wang, 2015; Guo et al., 2016; Tsai et al., 2016; Chang et al., 2017; Chueh et al., 2017; Yu et al., 2017; Becker et al., 2018; Li et al., 2018), one involved children (Becker et al., 2018), seven involved young adults (Giglia et al., 2011; Wang et al., 2013a, b; Jacobson and Matthaeus, 2014; Chang et al., 2017; Chueh et al., 2017; Yu et al., 2017), and six involved older adults (Dai et al., 2013; Huang C.J. et al., 2014; Tsai and Wang, 2015; Guo et al., 2016; Tsai et al., 2016; Li et al., 2018).

As an indication of how recently this topic has drawn investigator interest, 18 of these 19 articles were published after 2013. Collectively, within these 19 studies, a total of 1,845 participants were included. Study participants sample sizes ranged from 20 to 660, with an average sample size of 97 (*SD* = 140). The mean age of participants ranged from 9.6 to 70.5 years old. Across these 19 studies, a total of 21 cognitive tasks were used, measuring various aspects of cognitive function including inhibitory control, working memory, cognitive flexibility, planning, decision making, problem solving, processing speed, perception, attention, and memory. These characteristics of the intervention and observational studies are summarized in Tables 1, 2, respectively.

**TABLE 1:** Characteristics of the included intervention studies.

Study (Authors, Publication years, Methodological quality, Location)	N	OSE	CSE	Control group	Intervention duration/Session length	Cognitive tasks	Cognitive functions	Results
						
		(1) Gender	(1) Gender	(1) Gender				
		(2) Age (years)	(2) Age (years)	(2) Age (years)				
		(3) Exercise	(3) Exercise	(3) Exercise				
		(4) Education (years)	(4) Education (years)	(4) Education (years)				
**Children and adolescents (6–17 years old)**								
Crova et al., 2014 7/11 Italy	70	(1) 20M/37 (2) 9.6 ± 0.5 (3) Enhanced PE (including curricular PE classes and additional skill-based and tennis-specific training)	(1) 15M/33 (2) 9.6 ± 0.5 (3) Curricular PE	None	6 months	RNG task	Inhibitory control; Working memory	OSE led to greater improvements in inhibitory control compared with the CSE.
Schmidt et al., 2015 7/11 Switzerland	181	(1) 26M/69 (2) 11.3 ± 0.6 (3) Team games	(1) 28M/57 (2) 11.3 ± 0.6 (3) Aerobic exercise	(1) 28M/55 (2) 11.4 ± 0.6 (3) PE program with low physical exertion and cognitive engagement	6 weeks	N-back task; Flanker task	Inhibitory control; Cognitive flexibility; Working memory	OSE resulted in improvement on cognitive flexibility. CSE did not change cognitive function.
**Young adults (18–35 years old)**								
Hung et al., 2018 6/11 China (Taiwan)	20	(1) 20M/20 (2) 23.2 ± 2.5 (3) Badminton	(1) 20M/20 (2) 23.2 ± 2.5 (3) Running	None	40 min: 5 min (warm up) 30 min (exercise) 5 min (cool down)	Task-switching paradigm	Cognitive flexibility	One-bout OSE resulted in significantly higher serum BDNF and near significant smaller global switch costs compared with CSE.
**Older adults (≥ 56 years old)**								
O’Brien et al., 2017 5/11 Ireland	58	(1) 1M/18 (2) 69.2 ± 5.1 (3) Tennis, aerobics classes or dance classes	(1) 12M/19 (2) 69.2 ± 4.8 (3) Swimming or gym circuits, etc.	(1) 8M/21 (2) 70.5 ± 6.9 (3) Active retired group meeting or card games	OSE group: 80 ± 20 min; CSE group: 70 ± 20 min; Control group: 60 min	SiFI task; Forward Digit Span task	Memory (immediate memory); Multisensory perception	The immediate memory was improved in both exercise groups. Only OSE led to improvement in sensitivity in audio-visual perception.
Tsai et al., 2017 8/11 China (Taiwan)	64	(1) 22M/22 (2) 66.9 ± 4.7 (3) Table tennis (4) 12.5 ± 4.1	(1) 21M/21 (2) 66.2 ± 4.9 (3) Bike riding or brisk walking/jogging (4)12.6 ± 3.0	(1) 21M/21 (2) 65.7 ± 3.5 (3) A balance and stretching program (4) 10.6 ± 3.2	6 months	Task-switching paradigm; N-back task	Cognitive flexibility; Working memory	OSE and CSE differently influenced executive function. OSE led to improvement on cognitive flexibility. CSE led to greater improvement on working memory compared with the OSE.

*BDNF, brain-derived neurotrophic factor; CSE, closed skill exercise; M, male; OSE, open skill exercise; PE, physical education; RNG, random number generation; SiFI, sound induced flash illusion.*

**TABLE 2:** Characteristics of the included observational studies.

Study (Authors, Publication years, Methodological quality, Location)	N	OSE	CSE	Control group	Exercise experience	Cognitive tasks	Cognitive functions	Results
						
		(1) Gender	(1) Gender	(1) Gender	(1) Gender				
		(2) Age (years)	(2) Age (years)	(2) Age (years)	(2) Age (years)				
		(3) Exercise	(3) Exercise		(3) Exercise				
		(4) Education (years)	(4) Education (years)	(4) Education (years)	(4) Education (years)				
**Children and adolescents (6–17 years old)**								
Becker et al., 2018 10/12 The United States	660	(1) NA (2) Third grade (3) Baseball/softball; Martial arts; Hockey; Tennis; Football; Soccer; Basketball; Volleyball	(1) NA (2) Third grade (3) Swimming; Cheerleading; Track and field; Golf; Skateboarding; Dance; Skating	None	NA	Tower of Hanoi task	Inhibitory control; Cognitive flexibility; Working memory	There were no significant association of exercise modes (OSE and CSE) with executive function.
**Young adults (18–35 years old)**								
Chang et al., 2017 8/12 China (Taiwan)	60	(1) 15M/20 (2) 21.2 ± 1.2 (3) Martial arts training (4) 14.7 ± 0.9	(1) 14M/20 (2) 21.2 ± 1.8 (3) Marathon running (4) 15.0 ± 0.0	(1) 13M/20 (2) 21.6 ± 1.4 (3) Infrequent exercise/recreational activity (4) 14.8 ± 0.7	Martial arts: 8.6 ± 2.3 years; Marathon running: 7.8 ± 2.4 years; Control group: 0.9 ± 1.7 years	Stroop task; WCST; Tower of London task	Inhibitory control; Working memory; Cognitive flexibility; Planning	There were no differences in cognitive performance among the OSE, CSE and control group.
Chueh et al., 2017 8/12 China (Taiwan)	48	(1) 9M/16 (2) 20.0 ± 1.2 (3) Badminton or table tennis	(1) 9M/16 (2) 21.1 ± 2.3 (3) Swimming, triathlon, or distance running	(1) 9M/16 (2) 20.7 ± 1.1 (3) Sedentary control	OSE group: 10.8 ± 2.2 years; CSE group: 9.7 ± 3.2 years	Non-delayed and delayed match-to-sample test	Visuospatial attention; Visuospatial memory	The visuospatial attention and memory performance of the OSE and CSE groups were better than control group. There were no differences in cognitive function between OSE and CSE.
Giglia et al., 2011 8/12 Italy	56	(1) 23M/23 (2) NVP: 26.0 ± 4.3; RVP: 25.6 ± 3.4 (3) Volleyball	(1) 10M/10 (2) NR: 19.2 ± 4.0 (3) Rowing	(1) 10M/23 (2) 24.8 ± 2.5 (3) Sedentary control	NVP: 3.4 ± 1.0 hours/day; RVP: 1.1 ± 0.3 hours/day; NR: 3.1 ± 0.5 hours/day	Line-length judgment task	Visuospatial attention	Visuospatial attention was better in OSE group compared with the CSE and control group.
Jacobson and Matthaeus, 2014 7/12 The United States	54	(1) 14M/22 (2) 20.1 ± 1.2 (3) Externally paced exercise	(1) 3M/17 (2) 20.2 ± 1.5 (3) Self-paced exercise	(1) 6M/15 (2) 20.2 ± 1.3 (3) Sedentary control	Exercise group: ≥ 1 times/week	D-KEFS Tower test; D-KEFS Color-Word Interference Test; Coding test	Problem solving; Decision making; Inhibitory control; Processing speed	The problem solving and inhibitory control performance of the OSE and CSE groups were better than control group. The OSE group showed better problem solving compared with CSE group. The CSE group showed better inhibitory control compared with OSE group.
Wang et al., 2013a 9/12 China (Taiwan)	60	(1) 20M/20 (2) 20.7 ± 2.4 (3) Tennis	(1) 20M/20 (2) 19.3 ± 0.8 (3) Swimming	(1) 20M/20 (2) 20.4 ± 2.1 (3) Sedentary control	Tennis: 5.5 ± 2.8 years; Swimming: 4.9 ± 1.7 years	Stop-signal task	Inhibitory control	The OSE group showed better inhibitory control than the CSE and control group.
Wang et al., 2013b 7/12 China (Taiwan)	42	(1) 14M/14 (2) 20.6 ± 2.8 (3) Tennis	(1) 14M/14 (2) 19.4 ± 0.7 (3) Swimming	(1) 14M/14 (2) 21.2 ± 2.6 (3) Sedentary control	Tennis: 3–11 years; Swimming: 2.5–9 years	Go/No-Go Variable FP Paradigm	Decision making in inhibition	The OSE group showed better temporal preparation compared with control group. There were no differences between the OSE and CSE group.
Yu et al., 2017 9/12 China (Hong Kong)	54	(1) 10M/18 (2) 21.1 ± 2.2 (3) Badminton	(1) 11M/18 (2) 21.1 ± 2.0 (3) Track and field	(1) 9M/18 (2) 21.8 ± 2.1 (3) Sedentary control (no formal exercise training)	Badminton: 11.3 ± 2.7 years; Track and field: 7.9 ± 1.6 years	Task-switching paradigm; Simple reaction task	Cognitive flexibility; Processing speed	The OSE group had a lower switch cost of RT compared with CSE and control group when the task cue was 100% valid, whereas the OSE and CSE group had a lower switch cost of RT compared to the control group when the task cue was 50% valid. There were no differences in processing speed among the three groups.
**Older adults (≥ 56 years old)**								
Dai et al., 2013 8/12 China (Taiwan)	48	(1) 9M/16 (2) 69.0 ± 3.6 (3) Table tennis or tennis (4) 10.7 ± 2.8	(1) 6M/16 (2) 69.9 ± 3.6 (3) Jogging or swimming (4) 10.8 ± 4.2	(1) 2M/16 (2) 67.3 ± 3.0 (3) Irregular exercise (4) 13.0 ± 3.3	Table tennis/tennis: 13.0 ± 5.7 years; Jogging/swimming: 11.1 ± 4.5 years; Irregular exercise: 0.7 ± 0.6 years	Task-switching paradigm	Cognitive flexibility	The OSE and CSE group showed better cognitive flexibility compared with control group. The OSE group showed better cognitive flexibility compared with the CSE and control group.
Guo et al., 2016 8/12 China	111	(1) 17M/36 (2) 67.6 ± 5.9 (3) Table tennis (4) 12.6 ± 2.7	(1) 15M/38 (2) 66.7 ± 5.8 (3) Jogging or swimming (4) 11.4 ± 2.9	(1) 16M/37 (2) 66.9 ± 5.9 (3) Sedentary control (4) 11.0 ± 2.6	Exercise group: ≥ 30 min/session, ≥ 3 times/week, ≥ 1 year. Sedentary controls: inactivity or low activity level.	VWMT; VSMT; VMTT	Visuospatial working memory	The two exercise groups showed better performances on visuospatial working memory than the control group. The OSE group showed better performance on visuospatial short-term memory task than the control group. There were no differences in visuospatial mental rotation task among the three groups.
Huang C.J. et al., 2014 8/12 China (Taiwan)	60	(1) 11M/20 (2) 69.4 ± 3.0 (3) Table tennis, tennis, badminton, etc.	(1) 9M/20 (2) 70.6 ± 2.6 (3) Jogging, swimming, etc.	(1) 6M/20 (2) 68.3 ± 2.3 (3) Irregular exercise	OSE group: 7.8 ± 1.1 years; CSE group: 6.7 ± 2.4 years	Eriksen flanker task	Inhibitory control	The OSE and CSE group demonstrated better performance on inhibitory control compared with sedentary control group, whereas the OSE group showed better electrophysiological performance (i.e., event-related potential P300 amplitudes).
Li et al., 2018 8/12 China	75	(1) 15M/25 (2) 69.0 ± 3.4 (3) Table tennis or tennis (4) 10.7 ± 3.6	(1) 8M/25 (2) 69.8 ± 3.1 (3) Jogging or brisk walking (4) 11.2 ± 3.3	(1) 4M/25 (2) 67.8 ± 2.9 (3) Irregular exercise (4) 11.9 ± 3.4	Exercise group: ≥ 30 min/session, ≥ 3 times/week, ≥ 3 months.	SCWIT; Task-switching paradigm	Inhibitory control; Cognitive flexibility	The OSE and CSE group showed better performance on inhibitory control and cognitive flexibility compared with control group, while the OSE showed better electrophysiological performance (i.e., event-related potential smaller N200 and larger P300a amplitudes).
Tsai and Wang, 2015 8/12 China (Taiwan)	64	(1) 14M/21 (2) 65.4 ± 4.2 (3) Badminton or table tennis (4) 13.7 ± 3.0	(1) 14M/22 (2) 66.0 ± 4.1 (3) Jogging or swimming (4) 13.5 ± 3.5	(1) 13M/21 (2) 63.9 ± 3.4 (3) Sedentary control (4) 12.9 ± 2.0	Exercise group: ≥ 30 min/session, ≥ 3 times/week, ≥ 2 year.	Task-switching paradigm	Cognitive flexibility	The OSE and CSE group showed better performance on cognitive flexibility than control group. The OSE group showed better cognitive flexibility compared with the CSE and control group.
Tsai et al., 2016 8/12 China (Taiwan)	60	(1) 13M/20 (2) 65.3 ± 4.1 (3) Badminton or table tennis (4) 14.0 ± 2.8	(1) 14M/20 (2) 67.0 ± 4.7 (3) Swimming or jogging (4) 13.3 ± 3.6	(1) 13M/20 (2) 64.3 ± 3.6 (3) Sedentary control (4) 13.2 ± 2.0	Exercise group: ≥ 30 min/session, ≥ 3 times/week, ≥ 2 year. Sedentary controls: < 30 min/session, < 2 times/week, ≥ 2 year.	Central cue Posner paradigm	Visuospatial attention	The OSE and CSE group showed better performance on visuospatial attention than control group. The OSE could have more beneficial effects compared with CSE.

*CSE, closed skill exercise; D-KEFS, Delis–Kaplan executive function system; FP, foreperiod; M, male; NVP, national-level volleyball player; NR, national-level rowers; NA, not available; OSE, open skill exercise; RT, response time; RVP, regional-level volleyball player; SCWIT, Stroop Color-Word Interference Test; VMTT, visuospatial mental rotation task; VSMT, visuospatial short-term memory task; VWMT, visuospatial working memory task; WCST, Wisconsin card sorting test.*

### Methodological Quality of Included Studies

According to the PEDro scale, the average score of the methodological quality of the five intervention studies (Crova et al., 2014; Schmidt et al., 2015; O’Brien et al., 2017; Tsai et al., 2017; Hung et al., 2018) was 6.6, with scores ranging from 5 to 8 (see Supplementary Table 2 for details). The rating scores are also presented in Table 1.

Based on this 12-item assessment tool (Fuzeki et al., 2017; Engeroff et al., 2018), the average score of the methodological quality of the 14 observational studies was 8.1, with scores ranging from 7 to 10 (see Supplementary Table 3 for details). Thirteen (Giglia et al., 2011; Dai et al., 2013; Wang et al., 2013a, b; Huang C.J. et al., 2014; Jacobson and Matthaeus, 2014; Tsai and Wang, 2015; Guo et al., 2016; Tsai et al., 2016; Chang et al., 2017; Chueh et al., 2017; Yu et al., 2017; Li et al., 2018) of the 14 observational studies were found to be of “moderate quality” and one study (Becker et al., 2018) was judged to be of “high quality.” The rating scores are presented in Table 2.

### Study Findings

#### Observational Studies

Of the 14 observational studies, 12 (85.7%) showed that OSE group performed better on several aspects of cognitive function than the control group (Giglia et al., 2011; Dai et al., 2013; Wang et al., 2013a, b; Huang C.J. et al., 2014; Jacobson and Matthaeus, 2014; Tsai and Wang, 2015; Guo et al., 2016; Tsai et al., 2016; Chueh et al., 2017; Yu et al., 2017; Li et al., 2018). Nine studies found that both OSE and CSE group showed better performance of several aspects of cognitive function than the control group (Dai et al., 2013; Huang C.J. et al., 2014; Jacobson and Matthaeus, 2014; Tsai and Wang, 2015; Guo et al., 2016; Tsai et al., 2016; Chueh et al., 2017; Yu et al., 2017; Li et al., 2018). Furthermore, seven of 14 (50%) studies reported that the OSE group had better cognitive function compared with the CSE group (Giglia et al., 2011; Dai et al., 2013; Wang et al., 2013a; Jacobson and Matthaeus, 2014; Tsai and Wang, 2015; Tsai et al., 2016; Yu et al., 2017). The cognitive function measured in these studies included attention and executive function (i.e., inhibitory control, cognitive flexibility, and problem solving).

Only one observational study was conducted with participants who were children (Becker et al., 2018). This study showed that the two exercise modes (both OSE and CSE) were not significantly associated with performance of executive function (inhibitory control, working memory and cognitive flexibility) (Becker et al., 2018). Seven observational studies were conducted in young adults (Giglia et al., 2011; Wang et al., 2013a, b; Jacobson and Matthaeus, 2014; Chang et al., 2017; Chueh et al., 2017; Yu et al., 2017); and six (Giglia et al., 2011; Wang et al., 2013a, b; Jacobson and Matthaeus, 2014; Chueh et al., 2017; Yu et al., 2017) of these seven studies observed that the OSE group had better performances on inhibitory control, cognitive flexibility, problem solving, visuospatial memory, or visuospatial attention compared with the control group, while four showed that the OSE group had better cognitive performance in the domains of inhibitory control, visuospatial attention, problem solving or cognitive flexibility than the CSE group (Giglia et al., 2011; Wang et al., 2013a; Jacobson and Matthaeus, 2014; Yu et al., 2017). In contrast, Chueh et al. (2017) found that the cognitive performance (visuospatial attention and visuoapatial memory) of the OSE and CSE exercise groups was better than the control group, though the two exercise modes were not differently associated with the participants’ performance on cognitive function. Additionally, although a study by Chang et al. (2017) showed that participating in OSE and CSE was associated with improved physical fitness, this study found no significant difference in cognitive performance (executive function) among the three groups (OSE, CSE, and control group).

Of the 14 observational studies, the participants in six studies were adults older than 55 years (Dai et al., 2013; Huang C.J. et al., 2014; Tsai and Wang, 2015; Guo et al., 2016; Tsai et al., 2016; Li et al., 2018). Three of the six studies involving older adults (Dai et al., 2013; Tsai and Wang, 2015; Tsai et al., 2016) showed that OSE (versus the CSE and the control conditions) was more effective in enhancing performance on cognitive function (cognitive flexibility, or visuospatial attention). Guo et al. (2016) found that the OSE group demonstrated better performances on visuospatial working memory than the sedentary control group, but found no differences between the OSE and CSE groups. Meanwhile, both Huang C.J. et al. (2014) and Li et al. (2018) found that both OSE and CSE groups demonstrated better performance on executive function (i.e., inhibitory control and cognitive flexibility) compared with the sedentary control group, whereas only OSE group demonstrated a better electrophysiological performance (e.g., event-related potential P300 amplitudes).

#### Intervention Studies

Four (80%) intervention studies (Crova et al., 2014; Schmidt et al., 2015; O’Brien et al., 2017; Tsai et al., 2017) demonstrated that OSE led to improvements in some aspects of cognitive function (i.e., memory, audio-visual perception, cognitive flexibility, and inhibitory control), and three of these studies showed OSE to be superior to CSE for benefiting cognitive function (i.e., audio-visual perception, inhibitory control or cognitive flexibility) (Crova et al., 2014; Schmidt et al., 2015; O’Brien et al., 2017). Of the five intervention studies, two were conducted with children, with the exercise durations being 6 months and 6 weeks, respectively (Crova et al., 2014; Schmidt et al., 2015). The results consistently showed that OSE led to greater improvement of executive function (i.e., inhibitory control and cognitive flexibility) than CSE. Two intervention studies involved adults older than 55 years (O’Brien et al., 2017; Tsai et al., 2017), and one of these (Tsai et al., 2017) found a 6-month OSE intervention to yield improvements on executive function (i.e., cognitive flexibility) performance. In the same study, however, the CSE intervention resulted in better working memory performance compared with OSE (Tsai et al., 2017). The other study with older adults examined the acute effects of one-bout OSE and CSE intervention on cognitive function and found that immediate memory was improved in both exercise groups compared with control groups. The improvement of audio-visual perception was only found in the OSE group (O’Brien et al., 2017). Finally, one intervention study, using a crossover design, was conducted with young adults (Hung et al., 2018) and found that one-bout acute OSE led to a near significant trend of greater improvement in cognitive flexibility compared with CSE.

## Discussion

This systematic review critically evaluated the effects of OSE versus CSE on cognitive function. Collectively, we reviewed 19 study findings and found that 12 of 14 (86%) observational studies and four of five (80%) intervention studies supported cognitive benefits of OSE compared with control conditions. Furthermore, in seven of 14 (50%) observational studies and three of five (60%) intervention studies, participants in OSE groups had superior performance on several aspects of cognitive function compared with participants in CSE groups. Although the existing evidence tends to support that OSE may be more effective in benefiting some aspects of cognitive function (i.e., visuospatial attention, problem solving, audio-visual perception, inhibitory control, and cognitive flexibility) compared with CSE, it is premature to draw a clear picture on the effects of OSE versus CSE on a specific domain of cognitive function.

### Cognitive Benefits of OSE Versus CSE for Different Age Groups

Collectively, the findings of this systematic review suggested that the cognitive benefits of OSE versus CSE may vary across the developmental lifespan. Only three studies compared OSE versus CSE effects on cognitive function in children (Crova et al., 2014; Schmidt et al., 2015; Becker et al., 2018), and with the exception of the one observational study (Becker et al., 2018), the two intervention studies consistently demonstrated that the OSE intervention resulted in greater improvement of executive function than CSE (Crova et al., 2014; Schmidt et al., 2015). Previous studies suggested that the beneficial effects of physical exercise were more evident on executive function than on other aspects of cognitive function (Chaddock et al., 2011; Khan and Hillman, 2014). Evidence from the current review extends that impression from past literature by further suggesting that OSE may have superior benefits on executive function than CSE. Regular engagement in OSE likely stimulates brain regions that benefit brain development and executive function (Best, 2010). Thus, there are growing supports for integrating OSE into children’s exercise intervention programs, perhaps through physical education in school, as an effective means of promoting executive function (Crova et al., 2014; Schmidt et al., 2015).

With regard to young adults, although most of the included studies supported the beneficial effects of the two modes of exercise on cognitive function compared with sedentary counterparts, evidence for superior cognitive function benefits of OSE (versus CSE) is relatively limited, due to a scarcity of long term or “chronic” intervention studies. Four of the observational studies supported better cognitive performance in OSE (versus CSE) group participants (Giglia et al., 2011; Wang et al., 2013a; Jacobson and Matthaeus, 2014; Yu et al., 2017), but in the one intervention study (Hung et al., 2018), there was only near significant greater cognitive benefits resulting from the acute OSE (versus CSE) intervention. Therefore, the cognitive effects of OSE (versus CSE) in this age group are inconclusive. It is speculated that the limited evidence of the superior beneficial effects of OSE on cognitive function may be attributed to the fact that brain maturation and cognitive ability peak in young adulthood (Casey et al., 2000). Therefore, OSE cannot exert additional benefits on cognitive function in young adults. This review article also found that there were no existing studies involved middle-aged participants (aged 36–55 years). Future studies may consider this age group as potential participants.

In the older adults, evidence from this review’s six observational studies (Dai et al., 2013; Huang C.J. et al., 2014; Tsai and Wang, 2015; Guo et al., 2016; Tsai et al., 2016; Li et al., 2018) and two intervention studies (O’Brien et al., 2017; Tsai et al., 2017) consistently support a beneficial role of exercise on cognitive function. Furthermore, three observational studies (Dai et al., 2013; Tsai and Wang, 2015; Tsai et al., 2016) and two intervention studies (O’Brien et al., 2017; Tsai et al., 2017) suggested that, in this population, OSE may be more effective for improving attention, audio-visual perception, or cognitive flexibility. However, in this population as in others, it is worth noting that the beneficial effects of CSE (e.g., jogging) should not be neglected, even though there may be superior cognitive benefits for OSE.

Taken together, the results of the current systematic review indicate that OSE may be more effective in benefiting some aspects of cognitive function compared with CSE, especially in childhood and later adulthood. The findings not only help to clarify the differential cognitive effects of the two exercise modes, but also have some practical implications. For counteracting the prevalence of physical inactivity and sedentary behavior, it is reasonable to suggest that OSE should be incorporated into exercise promotion programs, as it may maximize the cognitive benefits of exercising.

### Potential Mechanisms of the Superior Effects of OSE Versus CSE

In this systematic review, the findings suggest a superior benefit of OSE for enhancing some aspects of cognitive function, perhaps especially in childhood and in late adulthood, as these two periods either precede the prefrontal lobe brain maturation that supports executive function (Casey et al., 2000), or are associated with an aging-related decline incognitive function. Of course, this is speculative, as the potential mechanisms underlying the superior effects of OSE over CSE remain unclear. OSE involves more cognitive loads and demands than CSE and this may partially explain its superior benefits in this systematic review. When performing OSE, participants are required to accommodate a continually changing environment. As such, there are greater cognitive demands and greater practice with some aspects of cognitive function that includes visuospatial ability, information-processing speed, multi-tasking flexibility, and other executive functions such as working memory and inhibitory control (Di Russo et al., 2010; Tsai et al., 2016, 2017). In contrast, CSE is performed in a predictable and stable environment in which participants are less likely to be exposed to multi-sensory stimuli than in OSE (Brady, 1995; Di Russo et al., 2010). CSE thus offers relatively less cognition guidance toward accomplishing a challenging goal or coordinating the body to execute complex movements (Di Russo et al., 2010; Tsai et al., 2016, 2017). Collectively, across the studies in this review, OSE came closer than CSE to satisfying theory that the cognitive demands and challenges of complex motor movement may be a pathway underlying the beneficial effects of exercise on cognitive function (Best, 2010). Additionally, social interaction that occurs during OSE training may exert a further positive impact on cognitive function (Best, 2010).

Physiologically, complex motor leaning and movement seems to exert longer positive influences on the neurotrophic system [i.e., the production of brain-derived neurotrophic factor (BDNF) and its receptor functioning] in the cerebellum than moderate-intensity running (Klintsova et al., 2004). BDNF plays a critical role in neural plasticity and is considered as a biomarker of exercise-induced cognitive benefits (Poo, 2001; Huang T. et al., 2014). A recent study in young adults also showed that one bout of OSE induced a greater increase in serum BDNF compared with a CSE intervention (Hung et al., 2018). Therefore, the greater neurophysiological changes that resulted from OSE may also support its superior cognitive benefits.

### Strength and Limitations

To the best of our knowledge, this was the first systematic review of the comparative effects of OSE versus CSE on cognitive function across the lifespan. Both intervention and observational studies were included in this review. In order to maximize between-study comparisons, we focused on studies that clearly defined the exercise modes based on a motor skill classification system yielding OSE and CSE categories. Despite the findings regarding cognitive benefits of both exercise modes (particularly to OSE) that we have outlined, the conclusions in this review must be considered within the context of its limitations. First, 14 of the 19 (74%) included studies were cross-sectional in design, and only five intervention studies were identified. These facts lend caution to making causal inferences. Yet, three of five (60%) included intervention studies supported a superior effect of OSE on some aspects of cognitive function compared with CSE, suggesting considerable value in further research pursuits. Second, we did not conduct a meta-analytic review due to the small number of randomized control trial (RCT) studies, the prevalence of diverse outcomes measures, and the wide age range of participants in these studies. Lastly, the search language we used was limited to English, increasing a risk of having omitted important research published in other languages.

## Conclusion

This review article systematically evaluated the current evidence of the effects of OSE versus CSE on cognitive function based on existing observational and intervention studies. The review tends to support the notion that OSE is superior in improving some aspects of cognitive function compared with CSE. Given that most of the existing studies are observational in design, with relatively few intervention studies, more rigorous RCTs with long-term follow-ups are needed to further confirm the current findings.

## Author Contributions

TH, QG, and LZ conceived the study. All authors contributed to the investigation process, provided the methodology, and wrote, reviewed, and edited the manuscript and approved its final version of the manuscript. QG and TH wrote the original draft of the manuscript. TH supervised the manuscript and acquired funding.

## Conflict of Interest Statement

The authors declare that the research was conducted in the absence of any commercial or financial relationships that could be construed as a potential conflict of interest.
