# Supplementary material for: Effects of Open Versus Closed Skill Exercise on Cognitive Function: A Systematic Review
Source: Front Psychol. 2019 Jul 27;10:1707. doi: 10.3389/fpsyg.2019.01707 (PMC6718477; doi:10.3389/fpsyg.2019.01707)
Supplement: Supplementary file 1 [file Data_Sheet_1.pdf]

Supplementary Table 1. Items of quality assessment tool for observational studies.

**Study purpose**
1. Was the study purpose clearly stated?
**Study design and methods**
2. Were eligibility criteria and the sources and methods of selection of participants clearly defined?
3. Were all outcomes, exposures, predictors, potential confounders, and effect modifiers clearly defined using standardized methods of acceptable quality?
4. Was exposure measurement carried out using standardized methods and measures and with acceptable quality?
5. Were the effects controlled for current (from physical activity assessment to cognitive function assessment) physical activity behavior?
6. Were the results adjusted for sedentary behavior?
**Statistical methods**
7. Was choice of confounders adjusted for, and in the case of subgroup analysis, was the definition of subgroups appropriate (sex, age, education or IQ, social surroundings, chronic diseases, alcohol, and smoking)?
8. Were all statistical methods, including those used to control for confounding and to examine subgroups and interactions, appropriate (i.e. sample size, statistical power)?
9. Were methods dealing with missing data appropriate?
**Results**
10. Were descriptive data and results of inductive analysis clearly stated?
11. Were unadjusted estimates and, if applicable, confounder-adjusted estimates and their precision (e.g., 95% confidence interval) given?
**Discussion**
12. Were study limitations clearly stated?

Supplementary Table 2. Quality assessment of intervention studies.

Study	Item 1	Item 2	Item 3	Item 4	Item 5	Item 6	Item 7	Item 8	Item 9	Item 10	Item 11	Sum Scores
**Children and adolescents (6-17 years old)**												
Crova et al., 2014	1	1	0	1	0	0	0	1	1	1	1	7
Schmidt et al., 2015	1	1	0	1	0	0	0	1	1	1	1	7
**Young adults (18-35 years old)**												
Hung et al., 2018	1	0	0	1	0	0	0	1	1	1	1	6
**Older adults (⩾ 56 years old)**												
O'Brien et al., 2017	1	0	0	0	0	0	0	1	1	1	1	5
Tsai et al., 2017	1	1	1	1	0	0	0	1	1	1	1	8

Note: Item 1 = eligibility criteria; Item 2 = randomization; Item 3 = concealed allocation; Item 4 = similar baseline; Item 5 = blinding of all subjects; Item 6 = blinding of all therapists; Item 7 = blinding of all assessors; Item 8 = more than 85% retention; Item 9 = intention to treat analysis; Item 10 = between-group comparison; Item 11 = point measures and measures of variability; "0" = absent or unclear; "1" = clearly described. A higher score indicates better methodological quality.

Supplementary Table 3. Quality assessment of observational studies.

Study	Item 1	Item 2	Item 3	Item 4	Item 5	Item 6	Item 7	Item 8	Item 9	Item 10	Item 11	Item 12	Sum Scores
**Children and adolescents (6-17 years old)**													
Becker et al., 2018	1	1	1	1	0	0	1	1	1	1	1	1	10
**Young adults (18-35 years old)**													
Chang et al., 2017	1	1	1	1	0	0	0	1	0	1	1	1	8
Chueh et al., 2017	1	1	1	1	0	0	0	1	0	1	1	1	8
Giglia et al., 2011	1	1	1	1	0	0	0	1	0	1	1	1	8
Jacobson and Matthaeus, 2014	1	1	1	1	0	0	0	0	0	1	1	1	7
Wang et al., 2013a	1	1	1	1	0	0	1	1	0	1	1	1	9
Wang et al., 2013b	1	1	1	1	0	0	0	1	0	1	1	0	7
Yu et al., 2017	1	1	1	1	0	0	1	1	0	1	1	1	9
**Older adults (⩾ 56 years old)**													
Dai et al., 2013	1	1	1	1	0	0	0	1	0	1	1	1	8
Guo et al., 2016	1	1	1	1	0	0	0	1	0	1	1	1	8
Huang C.J. et al., 2014	1	1	1	1	0	0	0	1	0	1	1	1	8
Li et al., 2018	1	1	1	1	0	0	0	1	0	1	1	1	8
Tsai and Wang, 2015	1	1	1	1	0	0	0	1	0	1	1	1	8
Tsai et al., 2016	1	1	1	1	0	0	0	1	0	1	1	1	8

Note: “0” = absent or unclear; “1” = clearly described. A higher score indicates better methodological quality.
